# Supplementary material for: Vertical Cylinder-to-Lamella Transition in Thin Block Copolymer Films Induced by In-Plane Electric Field
Source: Polymers (Basel). 2021 Nov 16;13(22):3959. doi: 10.3390/polym13223959 (PMC8622010; doi:10.3390/polym13223959)
Supplement: Supplementary file 1 [file polymers-13-03959-s001.zip › polymers-1444082-supplementary.pdf]

polymers

MDPI

Supplementary Materials

Article

Vertical Cylinder-to-Lamella Transition in Thin Block Copolymer Films Induced by In-Plane Electric Field

Alexey S. Merekalov [1], Yaroslav I. Derikov [1], Vladimir V. Artemov [2], Alexander A. Ezhov [1,3] and Yaroslav V. Kudryavtsev [1,4,*]

1 Topchiev Institute of Petrochemical Synthesis, Russian Academy of Sciences, 119991 Moscow, Russia; alexis@ips.ac.ru (A.S.M.); derikoff@ya.ru (Y.I.D.); alexander-ezhov@yandex.ru (A.A.E.)
2 Shubnikov Institute of Crystallography, Federal Scientific Research Centre "Crystallography and Photonics", Russian Academy of Sciences, 119333 Moscow, Russia; artemov@crys.ras.ru
3 Faculty of Physics, Lomonosov Moscow State University, 119991 Moscow, Russia
4 Frumkin Institute of Physical Chemistry and Electrochemistry, Russian Academy of Sciences, 119071 Moscow, Russia
* Correspondence: yar@ips.ac.ru

Distances between the nearest domains for different morphologies of the microphase-separated composite films

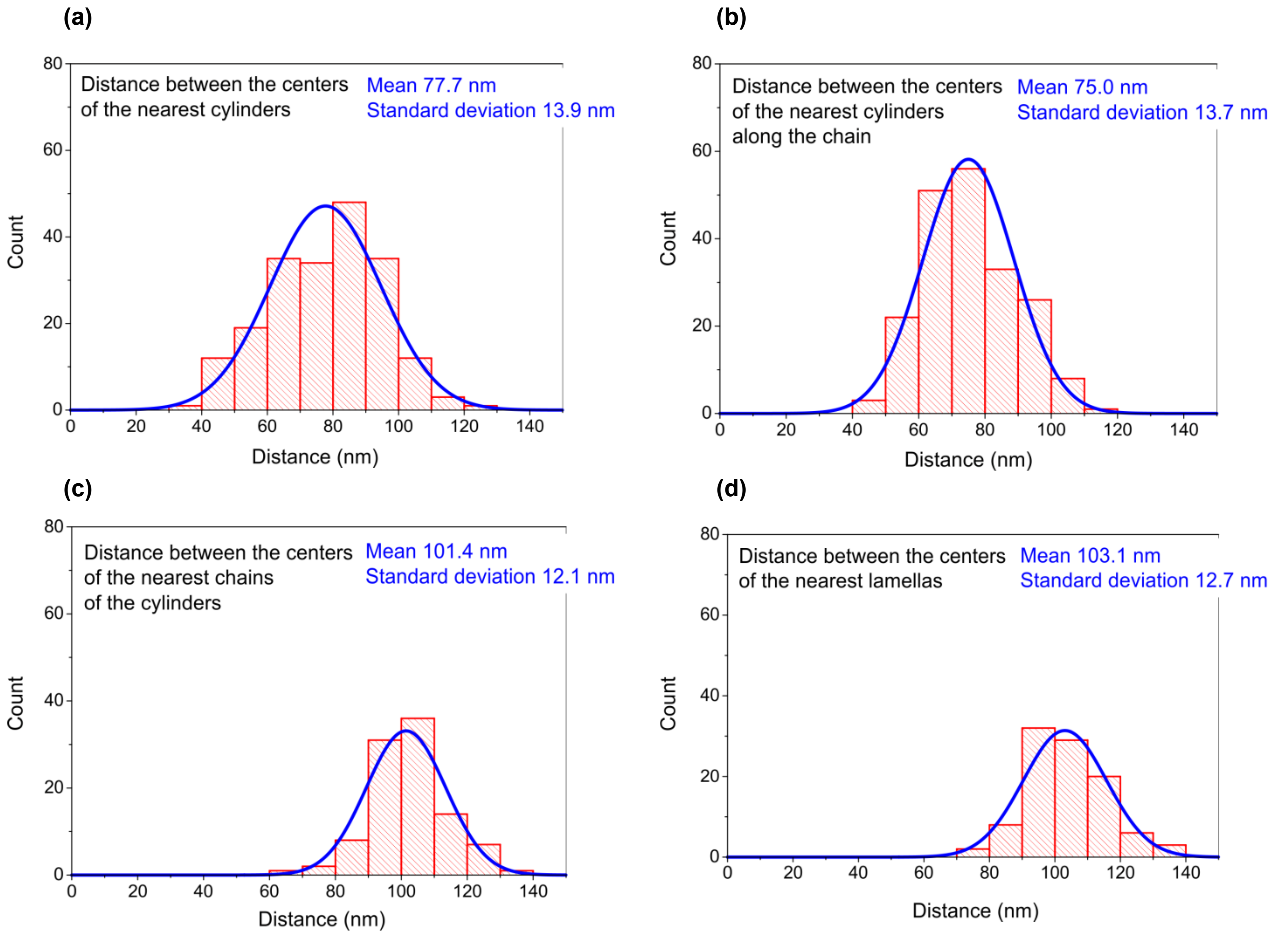

Figure S1. Characteristic distances in the microphase-separated PS–P4VP/AuNR composite films for the different morphologies: **(a)** hexagonally packed standing cylinders; **(b)** and **(c)** lined up cylinders; **(d)** standing lamellas formed upon cylinder merging.

Measurement of the microphase-separated block copolymer and composite films thickness using atomic force microscopy

To measure the thickness of a copolymer film, its surface was scratched. To this end, we used sample areas where the ITO layer was absent. In order to avoid damage to the glass substrate, the blade was made of copper. After the scratch was applied, an AFM study of its edges was carried out. The absence of a polymer film inside the scratch was established from (i) optical images obtained with an AFM-integrated video system (ii) AFM images showing the flat bottom of the scratch without any polymer structure features. Typical images of a scratch are shown in Fig. S2

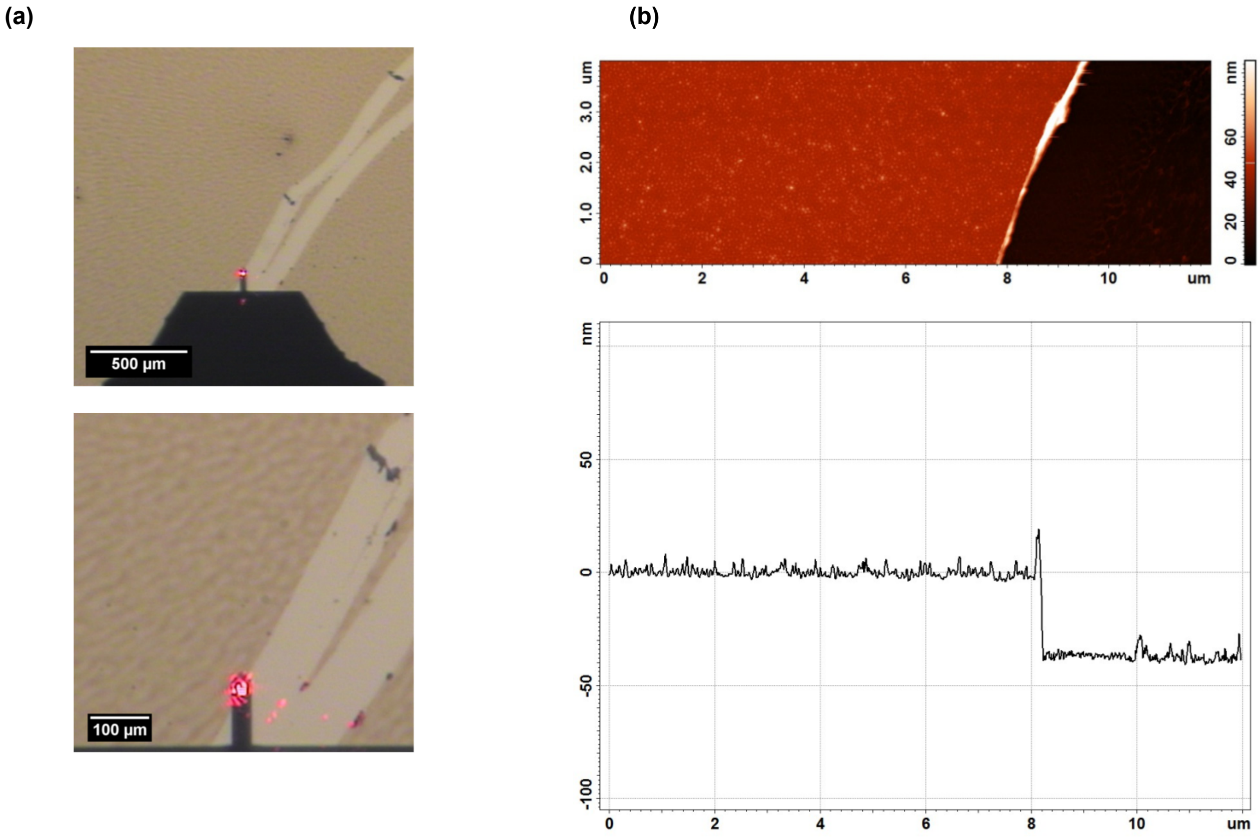

Figure S2. (a) Optical images of a scratch in the copolymer film. **(b)** The AFM image and cross-section of the scratch edge.

Scanning electron microscopy and energy-dispersive X-ray spectroscopy results obtained from the PS–P4VP/AuNR film annealed under chloroform vapor and electric DC field

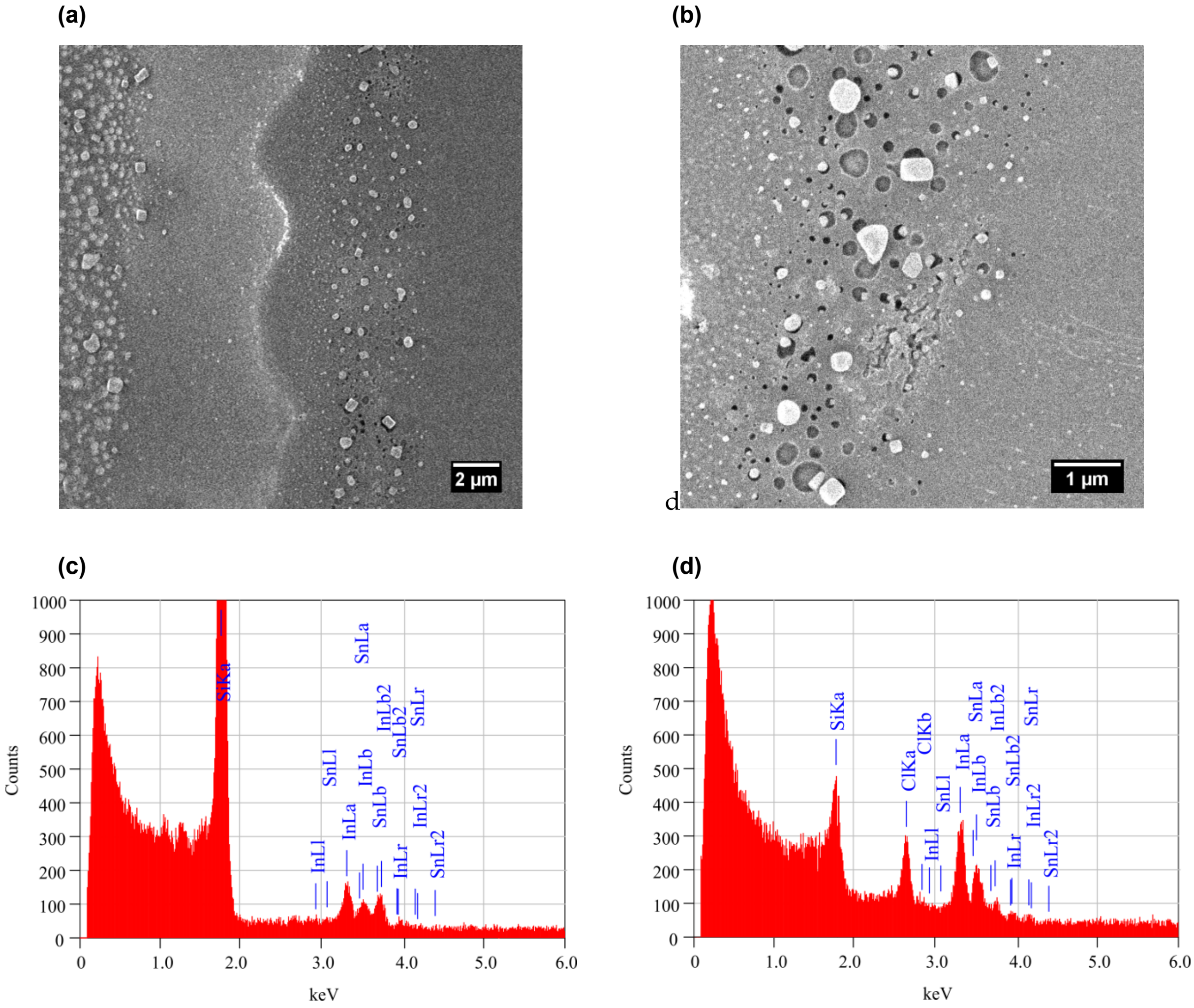

Figure S3. Scanning electron microscopy (SEM) images of the near indium tin oxide (ITO) cathode area and energy-dispersive X-ray *spectroscopy (EDS) results* obtained from the PS–P4VP/AuNR film annealed under chloroform vapor and electric DC field. **(a)** SEM images of the film deposited on ITO (left) and glass (right), (b) more detailed SEM image of the film deposited on glass near the ITO cathode, showing individual crystals of presumably indium chloride; (c) EDS spectrum averaged over the approx. 400 μm² area of the PS–P4VP/AuNR film deposited on the ITO film; (d) typical EDS spectrum of a single crystal formed during the film annealing.
